# Managing Postablation Pericardial Effusion

**DOI:** 10.19102/icrm.2018.091001

**Published:** 2018-10-15

**Authors:** Riccardo Cappato, Hussam Ali, Hugh Calkins, David Callans

**Affiliations:** ^1^Arrhythmia & EP Research Center, Humanitas Research Center & Humanitas University, Rozzano (Milan), Italy; ^2^Johns Hopkins Hospital, Baltimore, MD, USA; ^3^University of Pennsylvania Health System, Philadelphia, PA, USA

**Keywords:** Anticoagulation, atrial fibrillation, catheter ablation, pericardial effusion

## Drs. Cappato and Ali comment

Cardiac tamponade/perforation is still a major concern as a life-threatening complication following catheter ablation of atrial fibrillation (AF). Despite its typical occurrence in the acute setting, delayed cardiac tamponade has also been reported in up to 0.2% of patients after AF ablation and may lead to fatal outcomes.^1^

The early detection of pericardial effusion (PE) is crucial so that prompt management is ensured before hemodynamic collapse occurs. In many electrophysiology laboratories, the use of intracardiac or transesophageal echocardiography provides rapid recognition of such a complication. However, these imaging technologies may not be available in many centers due to logistical or economic issues. In practice, the fluoroscopic check of cardiac motion provides a useful tool for the early recognition of PE. As confirmed in animal studies and in our own experience, decreased cardiac silhouette excursion in the left anterior oblique projection is a sensitive and early sign of PE as soon as it is detectable by echocardiography.^2^

The current guidelines and expert consensus statements do not address how to manage minimal PEs that may be encountered not infrequently after AF ablation. Moreover, it is quite challenging to attribute such minimal PEs to an inflammatory process following ablation rather than to a direct traumatic injury. Knowing and understanding the clinical features, technical details of the ablation procedure, temporal course, and echocardiographic aspects of PE might be helpful in this context.

Though an exact mechanism of the delayed massive PE in the patient presented by Ziffra et al.^3^ is unknown, it might be multifactorial. The immediate formation, yet in a small amount, of PE at the end of the ablation procedure raises the suspicion of its traumatic rather than inflammatory nature. An initial traumatic mechanism (minor bruising) could have caused minimal PE, initiating an inflammatory process in the pericardial space. Subsequently, the oral anticoagulation therapy (OAT) might have worsened the clinical scenery, leading to a massive hematic PE.

Even in the era of reversal agents available for some nonvitamin-K anticoagulants (eg, idarucizumab for dabigatran), we generally use intravenous heparin in the immediate period postablation. In our center, on the following morning, if echocardiogram reveals stable minimal PE, oral anticoagulation is started under clinical surveillance. Echocardiography is repeated 48 hours after the procedure and under full-dose OAT. In the case of clinical and echocardiographic stability, the patient is discharged with a recommendation to undergo repeat echocardiography at one week later and is educated about warning symptoms for which he or she should seek prompt medical evaluation (eg, dyspnea, chest discomfort, fatigue, hypotension).

In this clinical setting, the current data do not favor the use of NOACs versus vitamin K antagonists or vice versa. However, the bridging period with heparin, typically used when starting vitamin K antagonists, might expose the patient to a brief period of excessive anticoagulation and an increased risk of bleeding. Furthermore, we do not routinely use colchicine or other anti-inflammatory drugs to manage asymptomatic minimal PEs. Further research is required to establish the optimal approach to manage minimal PEs after AF ablation regarding monitoring and follow-up, anticoagulation protocols, and anti-inflammatory therapy.

Riccardo Cappato, md, fesc, fhrs (riccardo.cappato@hunimed.eu)^1^ and Hussam Ali, md, fesc, fehra (hussam.ali@humanitas.it)^1^

^1^Arrhythmia & EP Research Center, Humanitas Research Center & Humanitas University, Rozzano (Milan), Italy

The authors report no conflicts of interest for the published content.

References1CappatoRCalkinsHChenSADelayed cardiac tamponade after radiofrequency catheter
ablation of atrial fibrillation: a worldwide report.J Am Coll Cardiol.2011582526962697
[CrossRef]
[PubMed]
2215295910.1016/j.jacc.2011.09.0282NanthakumarKKayGNPlumbVJDecrease in fluoroscopic cardiac silhouette excursion
precedes hemodynamic compromise in intraprocedural
tamponade.Heart Rhythm.200521112241230
[CrossRef]
[PubMed]
1625391310.1016/j.hrthm.2005.08.0023ZiffraJBGermanoNDPhillipsANOlshanskyBA case of postablation pericardial effusion.J Innov Cardiac Rhythm Manage.20189103365336810.19102/icrm.2018.091002PMC725266132477786

## Dr. Calkins remarks

In our practice, we use intracardiac echocardiography (ICE) during all AF ablation procedures, particularly to guide the transseptal puncture. A second benefit of ICE is that it allows for us to screen for a baseline effusion at the beginning of the case, prior to transseptal puncture, and also empowers us to be able to monitor for an effusion during the ablation procedure and prior to the sheaths being pulled at the end of the case. We do not routinely obtain additional echocardiograms during follow-up, unless a patient has signs or symptoms of an effusion/tamponade. It is well-established^1^ that delayed effusions may occur during a period of up to a month or more postablation, but their presentation is exceedingly rare. In the more than 20 years that I have been performing AF ablations, there have been only two patients who demonstrated an instance of “delayed effusion” resulting in symptoms.

As noted in the current case presentation by Ziffra et al.,^2^ it is very common to have a small/trace pericardial effusion immediately after ablation or on the following day. This generally results from the existence of inflammation associated with AF ablation. What is much less common to see are circumferential effusions more than 1 cm in size. Even if cardiac tamponade is not present, we have a low threshold to electively drain effusions of this size. However, this is very rare and I can recall only one or two cases of such over the past 20 years. Effusions ranging from trace to the 1-cm circumferential type noted above should be handled on a case-by-case basis and followed closely with serial echocardiograms.

In my opinion, the risk of a progressively enlarging effusion is no different on a novel oral anticoagulant (NOAC) than on Coumadin^®^ (warfarin; Bristol-Myers Squibb, New York, NY, USA). If anything, I would actually anticipate the risk to be lower on a NOAC. The Uninterrupted Dabigatran Etexilate in Comparison to Uninterrupted Warfarin in Pulmonary Vein Ablation (RE-CIRCUIT) trial^3^ demonstrated a dramatically lower risk of major bleeds with AF ablation performed on uninterrupted Pradaxa^®^ (Boehringer Ingelheim, Ingelheim am Rhein, Germany) as compared with warfarin.

One final comment: the way I see this case is that this patient potentially had significant pericarditis and a Dresslers-like syndrome, which resulted in her significant pericardial and pleural effusions. I believe that these effusions resulted from inflammation rather than bleeding. I agree completely with the approach to management, with the exception that I would not have stopped her anticoagulation for so long following her final pericardial and pleural drainage. As her CHA_2_DS_2_-VASc score was 4, stopping anticoagulation puts her at a significant risk of stroke.^4^

Hugh Calkins, md^1^

^1^Johns Hopkins Hospital, Baltimore, MD, USA

The author reports no conflicts of interest for the published content.

References1CappatoRCalkinsHChenSADelayed cardiac tamponade after radiofrequency catheter
ablation of atrial fibrillation: a worldwide report.J Am Coll Cardiol.2011582526962697
[CrossRef]
[PubMed]
2215295910.1016/j.jacc.2011.09.0282ZiffraJBGermanoNDPhillipsANOlshanskyBA case of postablation pericardial effusion.J Innov Cardiac Rhythm Manage.20189103365336810.19102/icrm.2018.091002PMC7252661324777863CalkinsHWillemsSGerstenfeldEPUninterrupted dabigatran versus warfarin for ablation in
atrial fibrillation.N Engl J Med.20173761716271636
[CrossRef]
[PubMed]
2831741510.1056/NEJMoa17010054CalkinsHHindricksGCappatoR2017 HRS/EHRA/ECAS/APHRS/SOLAECE expert consensus statement
on catheter and surgical ablation of atrial
fibrillation.Heart Rhythm.20171410e275e444
[CrossRef]
[PubMed]
2850691610.1016/j.hrthm.2017.05.012PMC6019327

## Dr. Callans discusses

The case report and discussion by Ziffra et al.^1^ illustrate a rare but potentially critical complication of ablation for AF. Late pericardial effusions are extremely uncommon following AF ablation. Cappato et al., using data from two worldwide surveys of AF ablation, noted 46 episodes of delayed cardiac tamponade in 27,921 procedures (incidence: 0.16% and mean presentation: 12 days after the procedure). Notably, most of the patients had nonspecific symptoms, but there were two deaths that occurred, while one patient experienced resuscitated cardiac arrest.^2^ In our clinic, we have previously observed late pericardial effusions (even in the complete absence of periprocedural effusion) that have been both reactive and bloody.

As the authors describe, we too perform all AF ablation procedures under ICE guidance and survey for complications including pericardial effusion at the end of the study. Until several years ago, we also performed routine transthoracic echocardiography the day after the ablation in order to confirm the absence of pericardial effusion; as expected, the yield of this was quite low.

If we detect a small effusion at the end of the procedure, we would monitor in the laboratory to ensure acute stability. In the absence of clinical symptoms or physical signs, I do not think we would manage these patients outside of our usual routine.

Given the (fortunately!) rare nature of this phenomenon, it is difficult to determine whether or not it is more likely to happen in the NOAC era or not. However, given the existing general observations about the NOAC effect and complications, I do not believe that NOACs contribute meaningfully to the risk of progressive effusion following AF ablation.

David Callans, md^1^

^1^University of Pennsylvania Health System, Philadelphia, PA, USA

The author reports no conflicts of interest for the published content.

References1ZiffraJBGermanoNDPhillipsANOlshanskyBA case of postablation pericardial effusion.J Innov Cardiac Rhythm Manage.20189103365336810.19102/icrm.2018.091002PMC7252661324777862CappatoRCalkinsHChenSADelayed cardiac tamponade after radiofrequency catheter
ablation of atrial fibrillation: a worldwide report.J Am Coll Cardiol.2011582526962697
[CrossRef]
[PubMed]
2215295910.1016/j.jacc.2011.09.028
